# Development of automated patch clamp assays to overcome the burden of variants of uncertain significance in inheritable arrhythmia syndromes

**DOI:** 10.3389/fphys.2023.1294741

**Published:** 2023-11-27

**Authors:** Joanne G. Ma, Jamie I. Vandenberg, Chai-Ann Ng

**Affiliations:** ^1^ Victor Chang Cardiac Research Institute, Darlinghurst, NSW, Australia; ^2^ School of Clinical Medicine, Faculty of Medicine and Health, University of New South Wales, Sydney, NSW, Australia

**Keywords:** cardiac electrophysiology, inheritable arrhythmia, automated patch clamp, *SCN5A*, *KCNH2*, *KCNQ1*, variant classification, functional genomics

## Abstract

Advances in next-generation sequencing have been exceptionally valuable for identifying variants in medically actionable genes. However, for most missense variants there is insufficient evidence to permit definitive classification of variants as benign or pathogenic. To overcome the deluge of Variants of Uncertain Significance, there is an urgent need for high throughput functional assays to assist with the classification of variants. Advances in parallel planar patch clamp technologies has enabled the development of automated high throughput platforms capable of increasing throughput 10- to 100-fold compared to manual patch clamp methods. Automated patch clamp electrophysiology is poised to revolutionize the field of functional genomics for inheritable cardiac ion channelopathies. In this review, we outline i) the evolution of patch clamping, ii) the development of high-throughput automated patch clamp assays to assess cardiac ion channel variants, iii) clinical application of these assays and iv) where the field is heading.

## Introduction

Cardiac myocyte action potentials, generated by the delicate balance of inward (*I*_Na_, *I*_Ca_, *I*_f_) and outward (*I*_KAch_, *I*_K1_, *I*_to_, *I*_Kur_, *I*_Kr_, *I*_Ks_) currents through the sarcolemma, are critical for coordinating the contractions of the myocardium (Grant, 2009). Disruptions, genetic or acquired, to ion channel function increase the risk of abnormal heart rhythms. Ion channelopathies arising from genetic variants in cardiac ion channel genes can lead to fatal arrhythmias without overt structural abnormalities in the heart. This is believed to be responsible for 40% of sudden cardiac deaths in young healthy individuals (Bagnall et al., 2016).

The discovery of cardiac ion channel genes underpinning Long QT Syndrome (LQTS; MIM 192500) in the 1990s–*KCNQ1* (K_V_LQT1) (Wang Q. et al., 1996), *KCNH2* (K_V_11.1/hERG) (Curran et al., 1995), *SCN5A* (Na_V_1.5) (Wang et al., 1995)—were a major breakthrough in our understanding of congenital arrhythmia syndromes. At least 20 genes have now been implicated in various inheritable arrhythmia syndromes, including LQTS, Brugada Syndrome (BrS; MIM 601144), Catecholaminergic Polymorphic Ventricular Tachycardia (MIM 604772), Short QT Syndrome (MIM 609620), Idiopathic Ventricular Fibrillation (MIM 603829), Progressive Cardiac Conduction Disease (MIM 113900), and Timothy Syndrome (MIM 601005). There is not however a simple relationship between diseases and ion channel mutations, with many patients showing oligogenic and polygenic inheritance patterns, whereby more than one genetic variant contributes to disease, and pleiotropy, where one gene can be responsible for different diseases (Cerrone et al., 2019).

The rapid evolution of genomic sequencing technology (van Dijk et al., 2014) and the introduction of high-throughput next-generation sequencing (Metzker, 2010) have made genetic testing more affordable and accessible. This has had clinical benefits for patients with genetic arrhythmogenic conditions, such as detecting or confirming diagnoses, guiding medical interventions and management, and aiding cascade screening (Musunuru et al., 2020). However, identifying a variant in a disease-associated gene does not necessarily mean that the variant is the cause of the disease in that individual. From large scale genome sequencing projects such as ExAC/gnomAD (Lek et al., 2016; Chen et al., 2022), it is now clear that rare missense variants occur in all individuals and the majority of these are benign. Thus, the American College of Medical Genetics and Association for Molecular Pathology (ACMG/AMP) developed guidelines to help interpret whether variants should be classified as pathogenic, benign or ‘Variants of Uncertain Significance’ (VUS) (Richards et al., 2015). Because most variants in rare diseases occur in a very small number of individuals it is often difficult to obtain sufficient clinical evidence to enable definitive evidence for pathogenicity on clinical data alone. Consequently, approximately half of all variants in genes implicated in cardiac diseases are classified as VUS (Anderson et al., 2022). Functional assays hold great promise to help ease the burden of VUS and appropriately calibrated functional assays can provide up to strong functional evidence for variant classification (Brnich et al., 2020). This is especially valuable for reporting secondary findings in medically actionable genes, of which 34/73 relate to cardiovascular phenotypes (Miller et al., 2021a).

There are numerous comprehensive reviews of inherited arrhythmia syndromes, including (Kapplinger et al., 2009; Kapplinger et al., 2010; Gray and Behr, 2016; Wilde and Amin, 2018; Schwartz et al., 2020; Wilde et al., 2022b; Remme, 2023). Here, we focus on recent developments in the functional characterization of genetic variants involved in inheritable arrhythmia syndromes.

## Automation of a gold-standard electrophysiological technique

### The patch clamp technique

In the 1940s, Hodgkin and Huxley proposed that ionic mechanisms underlie the initiation and propagation of neuronal action potentials (Hodgkin and Huxley, 1952; The Nobel Prize in Physiology or Medicine, 1963). The first cellular electrical recordings from heart tissues were performed in the late 1940s using microelectrodes (Coraboeuf, 1949; Coraboeuf and Weidmann, 1949), which set in motion the extraordinary progress that led to our understanding of the molecular mechanisms underlying cell electrophysiology and cardiac rhythms. The next big breakthrough occurred in the mid-1970s when Neher and Sakmann developed the patch-clamp technique that enabled the detection of single channel currents (Neher and Sakmann, 1976; The Nobel Prize in Physiology or Medicine, 1991). The patch clamp technique involves the formation of a giga-ohm seal between the cell plasma membrane and a glass micropipette containing electrolyte solution, thus isolating a membrane patch electrically (Hamill et al., 1981). The ion channels located in this membrane patch enable the movement of selected ionic currents into the micropipette for recording and determining conductance by an electrode. Variations of this technique have been used in electrophysiology studies, including *whole-cell* which detects the collective current across the entire cell membrane; *inside-out* where a cell patch is retracted into the bath solution allowing control of the cytoplasmic environment; and *outside-out* where conversely the cell patch is retracted and resealed, forming an independent patch facing the bath solution allowing control of the extracellular environment (Hamill et al., 1981). This technique remains the gold standard for *in vitro* electrophysiology however, it yields low throughput, even with a highly skilled experimentalist.

### Planar patch clamp

Over the last two decades there has been considerable interest in developing automated platforms to improve the throughput of patch clamp techniques (González et al., 1999). Ion channels play a crucial role in excitable tissues and are important therapeutic targets. In addition to screening for therapeutic efficacy, patch clamping has proven to be invaluable for toxicity studies and specifically screening for inadvertent drug block of hERG channels that can lead to potentially life-threatening cardiac arrhythmias (Fermini and Fossa, 2003). As such, considerable investments in automated patch clamp (APC) systems were driven by pharmaceutical companies.

**FIGURE 1:**
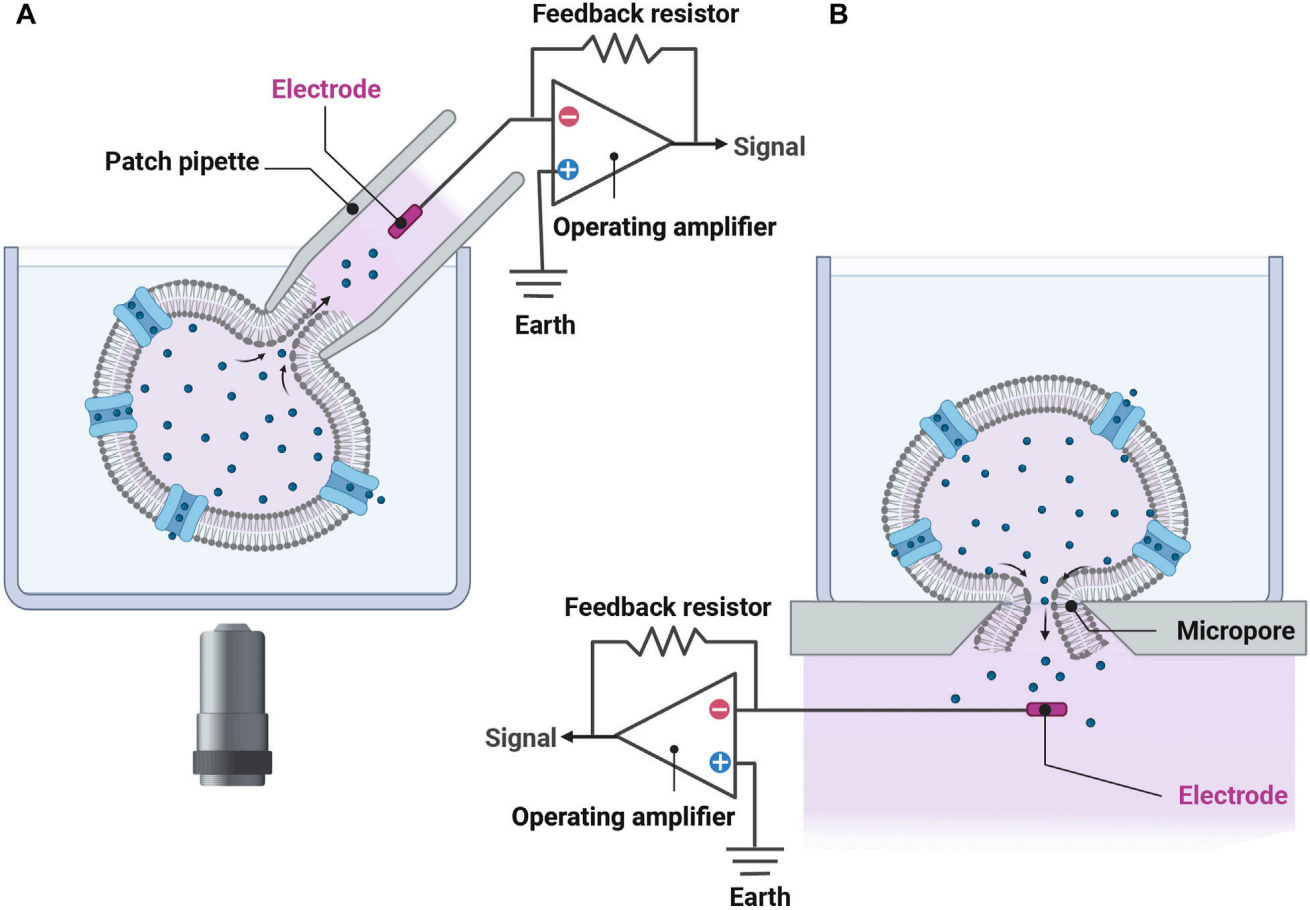
The transition from the conventional patch-clamp system to a planar patch-clamp system has enabled simultaneous patching of cells and underlies many of the APC used today. **(A)** In conventional whole-cell patch-clamp recordings, a pipette filled with electrolyte solution contains an electrode enabling the experimenter to apply various stimuli and measure the cell’s response. It takes considerable skill to attach the pipette to the cell using micromanipulators and a microscope. **(B)** In planar patch-clamp technology, cells are drawn down and adhered to a micropore using suction, acting similar to a pipette tip. Here, the cell is ruptured enabling stimulation and measurement of electrical activities. Chips used in modern APC platforms can record up to 768 cells in parallel (see Table 1). No micromanipulation is necessary. Figure developed using Biorender.

The pioneering development of the planar patch clamp, in 2002 (Fertig et al., 2002), allowed the simultaneous parallel patching of cells and is at the core of many APC used today. The use of a planar glass chip and suction below the chip to draw in the cell, forming a seal, then simultaneously rupturing the cell membrane to achieve electrical access to each cell in a 384-well format (Figure 1) can increase the output by two orders of magnitude compared to manual patch clamping. In addition to increasing throughput, APC offer many advantages including cell handling facilities, ability to clamp current or voltage, temperature control which is important for temperature-dependent channels and compounds, and internal solution exchange that enables activation or inhibition by compounds added intracellularly. Furthermore, APC is applicable to various cell expression systems used in cardiac electrophysiology experiments (Table 1), including human-induced pluripotent stem cell-derived cardiomyocytes (hiPSC-CMs) (Ma et al., 2011; Becker et al., 2013; Obergrussberger et al., 2016; Rajamohan et al., 2016; Li et al., 2019; Potet et al., 2020; Rapedius et al., 2022; Melgari et al., 2023). There are, however, some limitations. Most notably there is less consistency in high quality spatial and temporal voltage control which necessitates more stringent quality control. Failure to do this may result in significant variations in apparent *IC*_50_ values measured for the same drug on different days or different systems (Chambers et al., 2016; Kramer et al., 2020). The importance of stringent quality control is discussed in more detail below in reference to the analysis of gating defects in ion channel variants. A summary of the technical specifications of the latest APC systems available is shown in Table 1 and more in depth reviews of the application and history of APC can be found in Liu et al. (2019) and Bell and Fermini (2021).

**TABLE 1:** Various APCs have been developed over time, improving on prior models. The most current model of each series is detailed.

Instrument	QPatch 16X/48X	Qube	CytoPatch	IonFlux HT	Flyscreen 8,500	IonWorks Barracuda	PatchXpress	Patchliner	SyncroPatch 384/768 PE
Company	Biolin Scientific (Sophion)	Biolin Scientific (Sophion)	Cytocentrics	Fluxion Biosciences	Flyion GmbH	Molecular Devices	Molecular Devices	Nanion	Nanion
Year	2008	2014	2011	2010	2001	2013	2003	2006	2015
Preceded by	QPatch (2004)			Ionflux 16 (2009)		IonWorks (2002)		Port-a-Patch (2003)	SyncroPatch 96 (2009)
	QPatch HT (2006)					IonWorks Quattro (PPC) (2005)			
Recording substrate	Silicon	Polymer	Glass	PDMS	Glass	Polymer	Glass	Glass	Glass
Recording configurations	Whole cell	Whole cell	Whole cell	Whole cell	Whole cell	Perforated patch	Whole cell	Whole cell, cell attached, perforated patch, bilayer recording	Whole cell, perforated patch
No. Parallel recordings	8, 16 or 48	384	16–384	16 or 64	Up to 6	384	16	4 or 8	384 or 768
Throughput (data points/day)	250–3,000	30,000	200	2,500–8,000	“Several hundred”	1,100–6,000	1,500	250–500	20,000–38,000
Seal resistance	GΩ	GΩ	GΩ	GΩ	GΩ	50–100 MΩ	GΩ	GΩ	GΩ
Compatible cells	Cell lines, stem cells	Cell lines	Cell lines, primary, stem cell	Cell lines, primary cells	Cell lines	Cell lines	Cell lines, stem cells	Cell lines, primary, stem cells	Cell lines, stem cells
Temperature control	Yes	15°C–37°C	No	up to 40°C	No	No	No	Optional	Optional
Current clamp	Yes	Yes	Yes	No	No	No	Yes	Yes	Yes
Number of pipettes	2, 4 or 8	384	1	32	1–2	384	16	1	384
External solution exchange	Yes	Yes	Yes	Yes	No	Yes	Yes	Yes	Yes
Internal solution exchange	No	No	Yes	No	No	No	No	Yes	Yes
References	1, 2, 3	4	5, 6	7	8	9, 10, 11	12	13, 14	15, 16

1(Asmild et al., 2003).

2(Kutchinsky et al., 2003).

3(Mathes, 2006).

4(Chambers et al., 2016).

5(Scheel et al., 2011).

6(Stett et al., 2003).

7(Golden et al., 2011).

8(Lepple-Wienhues et al., 2003).

9(Finkel et al., 2006).

10(Gillie et al., 2013).

11(Schroeder et al., 2003).

12(Tao et al., 2004).

13(Xu et al., 2003).

14(Brüggemann et al., 2003).

15(Brüggemann et al., 2006).

16(Obergrussberger et al., 2016).

## Implementation of high-throughput APC assays to assess cardiac ion channel variants

Deleterious ion channel gene variants only impact their respective ionic current. Consequently, it is possible to assess the functional effect of ion channel variants in heterologous expression systems. Traditionally, these have employed manual patch clamp techniques (Bennett et al., 1995; Morais Cabral et al., 1998; Wang et al., 2000; Marx et al., 2002). More recently, APC platforms have been utilized for the characterization of large panels of ion channel variants (Vanoye et al., 2018; Glazer et al., 2020b; Kozek et al., 2020; Kuenze et al., 2020; Ng et al., 2020; Ng et al., 2021; Jiang et al., 2022; Ng et al., 2022; O’Neill et al., 2022; Vanoye et al., 2022; Ma et al., 2023; Thomson et al., 2023). APC has the potential to increase the throughput for variant functional assessment by two orders of magnitude compared to manual patch clamp. However, it is important to consider what effects of the variant can be assayed (e.g., Loss-Of-Function; LOF or Gain-Of-Function; GOF) and the potential sources of errors or challenges that can arise during these measurements when we design any high throughput APC assay.

### Protein trafficking

Trafficking-defective variants are the main cause of LOF in ion channel diseases. Distinguished by their minimal presence at the cell surface membrane, they are often caused by protein misfolding consequent to the inappropriate exposure of hydrophobic residues and subsequent aggregation with other misfolded proteins (Asher et al., 2006). Because of defective trafficking, these variants result in little or no assayable current during electrophysiological analyses (Delisle et al., 2004). As such, these variants are easily assayed in APC platforms. In the case of hERG channels, current density measured for a range of trafficking defective variants by APC, has been validated using ELISA assays (Ng et al., 2020), Western blot (Ng et al., 2022) and a massively parallel trafficking assay (Ng et al., 2022).

**FIGURE 2:**
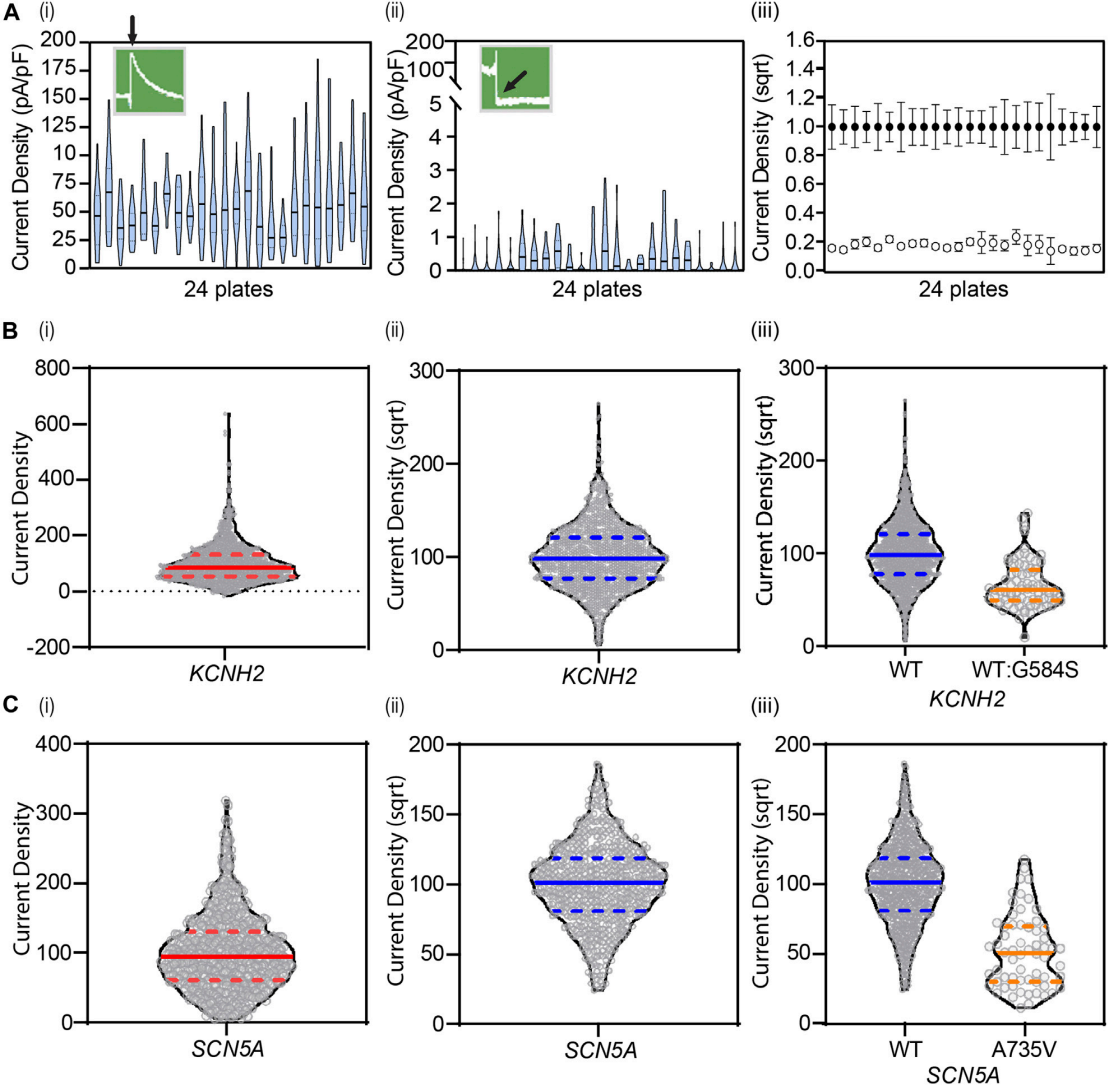
Cell-to-cell variability necessitates large numbers of replicate measurements to obtain an accurate estimate of the mean value. **(A)** Violin plots of *KCNH2* WT (i) and negative (ii) control lines show experimental cell-to-cell variability, plate-to-plate variability across 24 plates, and overall dynamic range of the assay revealing clear separation between positive and negative controls (iii). Data are shown as mean ± 95% CI. **(B)** Square-root transformations enable gaussian-distributed data for analysis as seen with *KCNH2* before (i) and after (ii) transformation (N = 1,198), and **(C)** *SCN5A* before (i) and after (ii) (N = 609). Transformations are applied to all data, including variants, enabling distinction of any changes in current density (iii). Solid lines denote median. Dashed lines denote quartiles. **(A)** Adapted from Jiang et al.(2022), with permission from Elsevier.

However, there is considerable variability in protein expression from cell-to-cell due to the stochastic nature of transcription and translation within each cell (Figure 2A) (Lachaud et al., 2022). Thus, it is necessary to undertake large numbers of measurements to ensure the average measurement is a true approximation of the population mean (Figure 2) (Jiang et al., 2022). In heterologous expression systems, there is the added complication that plasmids may be incorporated into random locations within the genome, and these may be associated with altered or inconsistent expression (Mizuguchi et al., 2000). This problem can be overcome by using a genomic landing pad for targeted gene expression, such as the Flp-In recombinase cassette, as this will ensure that all variant lines have the plasmid expressed in the same region of the genome (Ward et al., 2011). One can also utilize a tetracycline-induced expression element (Ng et al., 2021) to reduce the risk of the inserted plasmid being epigenetically silenced (Ríos-Pérez et al., 2021) and reduce the problem of constitutively expressed plasmids leading to reduced cell viability due to cell toxicity. The generation of stable cell lines does take longer (typically ∼3 weeks for antibiotic selection and expansion of positive clones) than using transient transfection (typically 48 h) but the reduction in variability between different transfections and being able to store stable lines for replicate experiments saves time and resources during data acquisition.

The application of Flp-In recombinase in high-throughput assays has been utilized by many researchers in a broad range of fields, including GPCRs (Ward et al., 2011) as well as sodium (Pablo et al., 2023), calcium (Pan et al., 2018; Baez-Nieto et al., 2022; Zhang et al., 2022) and potassium (Ng et al., 2021) ion channels. It is also possible to use IRES plasmids in combination with the Flp-In recombinase cassette so that two subunits, e.g., *KCNE1*+*KCNQ1* to recapitulate *I*_Ks_ or mutant + WT alleles to mimic heterozygous expression, are co-expressed from the same locus in all cells (Ng et al., 2021; Jiang et al., 2022). More recently, Jensen et al. (2020) have developed a double Flp-In system which when combined with IRES cassettes in theory could permit co-expression of 4 different subunits, although such an approach has not yet been published.

### Channel gating

The conformation of voltage-gated ion channels (e.g., closed, open, deactivated, inactivated and recovery states) are precisely regulated by membrane potential (Grant, 2009). Thus, to assess the impact of a variant that expresses sufficient current density (i.e., does not have a significant trafficking defect) require a thorough analysis of both the steady-state distribution between closed, open and inactivated states as well as the kinetics of transitions between these states. The specific protocols used will depend on the channel being studied and are typically outlined in the supplementary materials of the relevant papers (e.g., *KCNQ1*+*KCNE1* (Vanoye et al., 2018); *KCNH2* (Ng et al., 2020); *SCN5A* (Glazer et al., 2020b). Although less common, abnormal gating is still an important cause for both LOF and GOF variants that result in arrhythmia syndromes. For example, in K_V_11.1-N633S channels, enhanced inactivation can result in reduced current density (Ng et al., 2020) and in Na_V_1.5*-*R1632H channels, delayed recovery from inactivation reduces current density during standard depolarization steps (Glazer et al., 2020b).

**FIGURE 3:**
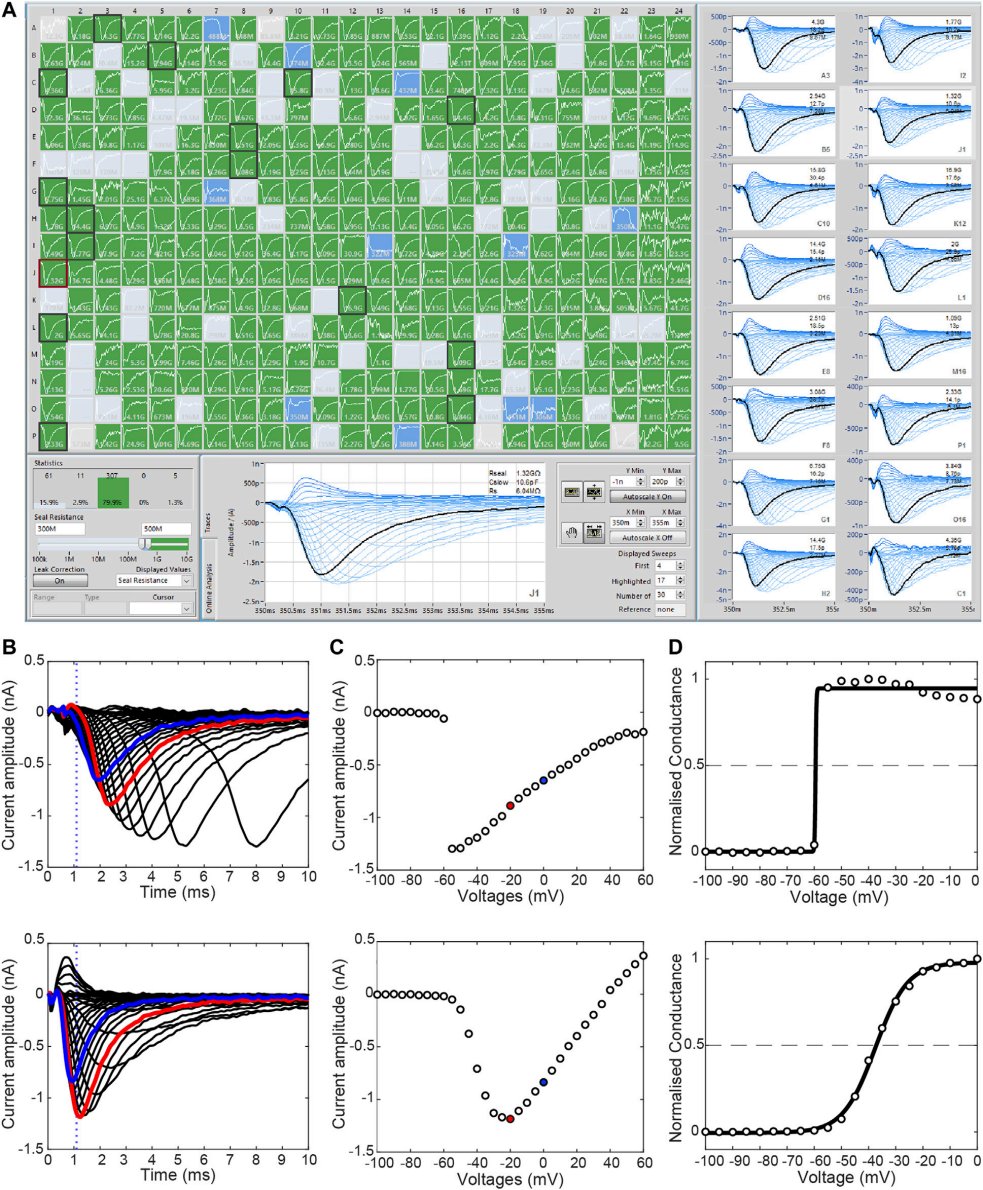
Stringent quality control is required to remove bad quality recordings. **(A)** The SyncroPatch 384 PE offers high throughput APC of mammalian cell lines. A minimum seal resistance of 500 MΩ is adequate to produce a high-quality seal (green wells). Cells with seal resistant <300 MΩ (grey) and between 300 and 500 MΩ (blue) indicate lower quality seals. **(B)** Current traces illustrating the activation of two WT SCN5A HEK293 cells (−100 mV to +60 mV, Δ5 mV). The top row shows an example of current recorded with poor voltage control whilst the bottom row shows recordings from a cell with good quality voltage control. Due to the rapid kinetics of sodium channel, a “time to peak” measurement can be used to identify poorly clamped cells. In this instance, time to peak is applied at 0 mV (blue) and dotted line represents 1.1 ms post-activation. **(C)** Respective current-voltage curves revealing steep jump in current in the poorly clamped cell (top). **(D)** Steady-state activation and Boltzmann function of respective cells reveal the poorly clamp cell (top) have visibly severely impacted V_50_ (voltage required to activate half of the cells’ channels) and slope, which is typically used for comparison between cells. Red indicates −20 mV sweep used for current density measurements in this case.

The accuracy of the measurement of voltage-dependent gating parameters relies heavily on the quality of temporal and spatial voltage clamp during recordings (Hamill et al., 1981). The quality of the voltage clamp is influenced by the size of the currents being recorded, i.e., the larger the current the more difficult it is to ensure effective spatial and temporal control of voltage. Experimentally, one way to limit this problem is to adjust ion concentrations in the recording solutions to reduce the driving force and consequently amplitude of current. The accuracy of voltage clamp control is also critically dependent on the access resistance between the cell and the recording patch electrode or across the ‘hole’ in a planar patch clamp recording. There is a positive correlation between access resistance values and voltage errors in patch clamp measurements. For example, when measuring *I*_Na_ with current amplitudes of 10 nA, compared to current amplitudes of 1 nA, series resistances of 2 and 5 MΩ have been demonstrated to shift V_50_ of activation by −7 and −11 mV and reduce the slope factor by a factor of 1.5 and 1.8, respectively (Montnach et al., 2021). It is therefore recommended to use low access resistance plates for APC recordings. However, this needs to be balanced against the fact that if the holes are larger (therefore giving a lower access resistance) it is harder to achieve high quality seals, especially with smaller cells. Also, given that the changes to gating kinetics caused by a variant will be similar irrespective of the level of current expressed in each cell, it is advisable to apply more stringent quality control criteria (Figure 3; e.g., minimum seal resistance, maximum series resistance, time to peak current, and minimum and maximum current amplitudes) for the analysis of gating characteristics to produce more reliable data (Rapedius et al., 2022).

### Ion selectivity

Changes in ion selectivity can be assayed by measuring differences in reversal potential between variants and WT channels. Such measurements do not enable precise quantification of the change in ion selectivity but are generally sufficient to identify that it will cause abnormal function (Ng et al., 2020). As with measuring gating defects, it is also important to ensure you have high quality voltage clamp control when measuring reversal potentials.

### Combined defects

Some trafficking-defective proteins result in only partially reduced expression at the cell surface. These proteins may also have abnormal gating (Gui et al., 2010; Balijepalli et al., 2012; Kanters et al., 2015; Perry et al., 2016), which may exacerbate the effect of the trafficking defect or in some cases reduce the impact of the gating defect. Thus, it is sensible to design protocols that assess current density under physiologically-relevant voltage ranges that can provide an integrated assessment of both trafficking and gating defects. For example, *I*_Kr_ tail current density at −50 mV (peak current during repolarization in cardiac myocytes) after a 1s depolarization step to +40 mV (to mimic cardiac action potential) will take into account the effect of trafficking defects, slow or reduced activation during the depolarization step and enhanced inactivation at −50 mV during the repolarization phase. Similarly for *I*_Na_, the peak current density measured during a depolarization step from a holding potential of −90 mV (to mimic the cardiac resting membrane potential) can reveal reduced channel availability due to hyperpolarized inactivation that would otherwise have not been observed when using a holding potential of −120 mV. For example, Ma et al. showed that *SCN5A* variants E1784K, R1632H and R1632C produce a more marked reduction in current density when holding cells at −90 mV compared to −120 mV relative to what is seen for WT channels (Ma et al., 2023) concordant with previous manual patch clamp studies for E1784K (Makita et al., 2008), R1632H (Benson et al., 2003), and R1632C (Nakajima et al., 2015). An alternative approach is to use parameters derived from the analysis of steady-state activation/inactivation and kinetics of gating transitions to adjust *in silico* ion channel models that can then be incorporated into *in silico* models of the cardiac action potential (Clancy et al., 2003; Zhao et al., 2009; Romero et al., 2015; Kernik et al., 2020; Aghasafari et al., 2021).

## APC assays for arrhythmogenic ion channelopathies: current approaches and challenges

The most common cardiac ion channelopathy is the Long QT Syndrome (LQTS), which is characterized by a prolonged QT-interval on the surface electrocardiogram (Roden, 2008). LQTS is associated with early after depolarizations and *Torsades de Pointes*, a form of polymorphic ventricular tachycardia that leads to ventricular fibrillation and sudden cardiac death. According to the latest guidelines (Adler et al., 2020), *KCNQ1*, *KCNH2* and *SCN5A* are the only ion channel genes definitively associated with LQTS. In addition, *KCNQ1* and *KCNH2* are the only two ion channel genes definitively associated with Short QT Syndrome, and *SCN5A* is the only gene definitively implicated in Brugada Syndrome (BrS). To date, specific APC assays have been developed for assessing the effect of genetic variants found in *KCNQ1*, *KCNH2* and *SCN5A*.

### KCNQ1

The cardiac voltage-gated potassium channel K_V_7.1, encoded by *KCNQ1*, consists of four identical pore-forming subunits, each with six transmembrane helices and a pore loop. It co-assembles with the β-subunit *KCNE1* to form the channel complex that passes the slow delayed rectifier potassium current (*I*_Ks_) in the heart (Barhanin et al., 1996; Sanguinetti et al., 1996). *KCNE1* is essential in modulating the slow activation kinetics of *I*_Ks_. The *KCNQ1*-*KCNE1* complex also does not inactivate on the timescale relevant to cardiac repolarization, when compared to channels encoded by *KCNQ1* alone (Pusch et al., 1998). *KCNQ1*-*KCNE1* channels were the first ion channel investigated using the SyncroPatch 384 PE APC system (Vanoye et al., 2018). CHO cells were doubly transfected with *KCNQ1* plasmids (either WT or one of 78 variants) coupled to GFP and *KCNE1* plasmid coupled to RFP. Transfected cells were then quantified using flow cytometry to determine transfection efficiency, before being analyzed for current density and voltage-dependence of activation. As LQTS is an autosomal dominant condition, a subset of 56 *KCNQ1* variants (coupled to eGFP) were co-transfected with WT *KCNQ1 (*coupled to mScarlet), in a CHO cell line stably expressing *KCNE1,* to investigate possible dominant-negative effect of variants. The authors used the data from this assay as strong evidence for pathogenicity, providing evidence for the reclassification of 23/35 VUS as likely pathogenic (Vanoye et al., 2018). The application of this assay (as well as *KCHN2* and *SCN5A* assays) in the comprehensive eMERGE-III sequencing study of participants without known cardiac indications provided evidence to support the reclassification of 5/30 VUSs tested *in vitro* (Glazer et al., 2022).

#### Limitation

β1-Adrenergic receptor (β1-AR) activation impacts intracellular cAMP levels leading to protein kinase A (PKA) activation, resulting in phosphorylation of the N-terminal region of *KCNQ1*. This leads to faster channel activation and, ultimately, shortening of the cardiac action potential (Dixit et al., 2020). Explicitly, LQTS-associated *KCNQ1* variants can prolong the action potential duration disruptions to the rate-dependent shortening typically instigated by β-adrenergic stimulation. Assaying β-adrenergic regulation of *I*_Ks_ in heterologous systems however is not straight forward as it requires co-expression of *AKAP9* (Yotiao) scaffolding protein in the heterologous expression system (Marx et al., 2002; Dvir et al., 2014). A-kinase anchoring protein 9 (AKAP9) binds to the regulatory subunit of PKA, protein phosphatase 1, phosphodiesterase and adenylate cyclase; thereby providing the scaffold for adrenergic regulation of *I*_Ks_ function (Marx et al., 2002; Kurokawa et al., 2003). To date, no APC assays incorporating AKAP9 have been developed, thus, one cannot be certain that variants with a normal *I*_Ks_ current density in these assays do not affect β-adrenergic regulation.

### KCNH2

The cardiac voltage-gated potassium channel K_V_11.1, encoded by *KCNH2*, contains four identical pore-forming subunits with 6 transmembrane helices. In addition, it has large cytoplasmic PAS domain at its N-terminal and cyclic nucleotide binding homology domain at its C-terminal (Vandenberg et al., 2012), which are both important for maintaining the protein stability needed for trafficking (Ke et al., 2013; Anderson et al., 2014), and regulation of the slow channel activation, fast inactivation and slow deactivation (Morais Cabral et al., 1998; Wang et al., 1998; Aydar and Palmer, 2001; Vandenberg et al., 2012; Ng et al., 2014). The current passed by K_V_11.1 potassium channels is known as the rapid delayed rectifier potassium current, *I*_Kr_. Loss of *I*_Kr_ can be due to four mechanisms: reduced synthesis, defective trafficking, defective gating, or altered ion permeation (Delisle et al., 2004). Defective protein trafficking is the principle underlying cause of *KCNH2*-related long QT syndrome (Anderson et al., 2014). In the first APC assay for *KCNH2* variants, the function of 23 homozygous *KCNH2* variants were first assessed and compared to expression measured using an ELISA assay (Ng et al., 2020). This was followed with an analysis of 30 heterozygous *KCNH2* variants expressed using bicistronic plasmid to assess the dominant-negative effect in stably expressed Flp-In HEK293 cells (Ng et al., 2020). The use of stably integrated doxycycline-inducible Flp-In HEK293 allowed the isogenic comparison between the functional effect of variant and WT to prevent random insertion thereby ensuring reliability in current density measurement. The use of stable cell lines also allowed generation of a biobank of *KCNH2* variant cell lines (Ng et al., 2021; Ng et al., 2022). Current density of *KCNH2* variants quantified by APC in heterozygous expression system, when sufficiently large N numbers were obtained, not only represents the trafficking phenotype when it was compared to ELISA (Ng et al., 2020), but also allowed for assessment of gating defects and changes to ion permeability (Ng et al., 2020).

This *KCNH2* assay has also been used to cross validate a massively parallel trafficking assay for *KCNH2* (Kozek et al., 2020). In a follow up study, Ng et al. (2022) investigated 458 single-nucleotide missense variants in exon 2 of *KCNH2*, the largest APC experiment performed to date. The APC data generated for these 458 *KCNH2* variants was used as the benchmark for validating an independently acquired massively parallel trafficking assay dataset for exon 2 of *KCNH2* (Ng et al., 2022), a known hotspot for causing *KCNH2*-related long QT syndrome. In this study 42% of variants showed >50% reduction in protein trafficking, of which 65% exerted a dominant negative effect when co-expressed with WT (Ng et al., 2022). A separate massively parallel trafficking assay dataset for the S3 to S5 transmembrane helices has also been generated, which included 51 clinically identified variants, and the trafficking results for them were cross-validated against the peak tail current density data measured by the *KCNH2* assay, which found that 4/6 were correctly classified as functionally normal and 42/44 correctly classified as LOF (Ullah et al., 2022).

Before these assays can be used to provide evidence to support classification of variants as pathogenic or benign, it is essential that they are formally assessed according to the criteria published by the ClinGen Sequence Variant Interpretation (SVI) Working Group (Brnich et al., 2020). To date, the *KCNH2* APC assay is the only assay that has been formally calibrated using clinically-verified pathogenic and benign variant controls to determine the sensitivity and specificity of the assay (Jiang et al., 2022). From the performance of the assay, the odds of pathogenicity of the *KCNH2* assay was determined to have the equivalent strength of moderate evidence level for both normal and abnormal protein function, enabling the reclassification of 16% of VUSs assessed as likely pathogenic (Jiang et al., 2022). Recently, this assay has been updated to provide strong evidence strength using 30 benign and 30 pathogenic variant controls (Thomson et al., 2023).

### SCN5A

The cardiac voltage-gated sodium channel Na_V_1.5, encoded by *SCN5A*, contains four pore-forming domains, but unlike the cardiac potassium channels, these four domains are part of a single polypeptide (de Lera Ruiz and Kraus, 2015). Typically, LOF mutations in *SCN5A* result in BrS, whereas gain of function mutations results in LQTS. However there are many other syndromes associated with *SCN5A* variants including sudden infant death syndrome, dilated cardiomyopathy, progressive cardiac conduction disorder, sick sinus syndrome, atrial fibrillation, early repolarization syndrome, and idiopathic ventricular fibrillation (Amin et al., 2010; Moreau et al., 2015; Gray and Behr, 2016; Liu et al., 2016; Remme, 2023). Moreover, some *SCN5A* variants can cause mixed phenotypes, referred to as “sodium channel overlap syndrome” (Bezzina et al., 1999; Makita et al., 2008; Wilde and Amin, 2018). Loss of sodium channel activity results in decreased cardiac excitability and reduced electrical conduction velocity, thereby increasing the risk of cardiac arrhythmias (Wilde and Amin, 2018). LQTS3, the second most prevalent *SCN5A*-associated disease (accounts for 5%–10% of LQTS patients) is caused by gating-defective *SCN5A* variants that cause GOF via a loss of inactivation (Liu et al., 2016; Wilde and Amin, 2018). The resulting increased late *I*_Na_ delays repolarization which leads to the prolongation of the QT interval on the surface ECG.

The first APC assay for *SCN5A* variants incorporated an mCherry-blasticidinR fusion protein, forming *SCN5A*:IRES:mCherry:blasticidinR (Glazer et al., 2020a; Glazer et al., 2020b). This enabled the selection of blasticidin-resistance present on the *SCN5A* plasmid and quantification through flow cytometry for mCherry-positive cells (or Blue Fluorescent Protein/iCasp for non-integrated cells) to determine the proportion of cells with successful plasmid integration. Using this *SCN5A*-BrS APC assay, the data supported reclassification of 61/83 VUS (Glazer et al., 2020b). The feasibility in applying this approach in the clinical setting was recently demonstrated in the eMERGE-III for VUS reclassification (Glazer et al., 2022). More recently, Ma et al. (2023) have optimized an *SCN5A* APC assay for assessing LOF in BrS-associated variants that satisfies the recommendations from the ClinGen SVI Working Group (Brnich et al., 2020) to establish clinical grade evidence for assistance with classification of VUS. The odds of Pathogenicity (OddsPath) scores for this assay indicate that the assay can achieve strong evidence levels for both normal (BS3) and abnormal (PS3) protein function (Ma et al., 2023).

On the other end of the disease spectrum, an *SCN5A*-LQTS (GOF) assay is not yet available. A major reason for this is the very small size of the late sodium current (approximately 0.2% of peak of WT). Thus, it is not possible to design a single assay that can record both GOF and LOF, as the high Na^+^ concentrations required to measure GOF in the late sodium current (Wang D. W. et al., 1996; Keller et al., 2003; Tester et al., 2010; Ma et al., 2023; Stutzman et al., 2023) will generally create voltage clamp errors when trying to measure peak sodium currents. GOF defects are primarily a result of failures in channel inactivation resulting in a late/persistent current (Wang D. W. et al., 1996; Keller et al., 2003; Ruan et al., 2007; Makita et al., 2008; Olesen et al., 2012; Moreau et al., 2013; Peters et al., 2016; Veltmann et al., 2016; Li et al., 2021; Stutzman et al., 2023) or caused by a change in the overlap of the voltage dependence of steady-state activation and inactivation resulting in a window current (Wang D. W. et al., 1996; Moreau et al., 2013; Moreau et al., 2015; Peters et al., 2016; Peters et al., 2021; Stutzman et al., 2023). Additional mechanisms that can contribute include faster recovery from inactivation (Clancy et al., 2003) and the presence of gating pore currents (Moreau et al., 2015; Peters et al., 2021). Thus, any APC assay designed to detect *SCN5A* GOF will have to be able to assay these diverse range of mechanisms.

#### Limitation

Though traditionally thought to form functional monomers, dominant-negative effects in Na_V_1.5 have been reported *in vitro* (Keller et al., 2005; Hoshi et al., 2014; O’Neill et al., 2022) and *in vivo* (Doisne et al., 2021). Interactions between Na_V_1.5 α-subunits was suggested to cause dominant-negative effects observed in trafficking-defective (Clatot et al., 2012), and gating-defective variants (Clatot et al., 2018). They corroborated this with co-immunoprecipitation, protein crosslinking, western blots, single molecule pull-down and electrophysiological analysis (Clatot et al., 2012; Clatot et al., 2017). However, naturally, Na_V_1.5 proteins form macromolecular complexes with Na_V_1.5-interacting proteins that can modulate its trafficking and gating (Marchal and Remme, 2022). The impact of *SCN5A* variants on these interactions are not yet clear but, altered protein-protein interactions within Na_V_1.5 complexes have been associated with GOF and LOF defects, predominantly at the intercalated discs and lateral membrane regions, respectively. Additionally, variants in the interacting-proteins (e.g., Na_V_1.5 β-subunits, Ankyrin-B, Caveolin-3) have also been associated with sodium channelopathies. Hence, whether these dominant-negative effects are truly dominant-negative or are a result of altered subdomain-specific Na_V_1.5-interacting proteins will require further investigation.

## Broader limitations of APC functional genomics assays

Beyond the channel-specific limitations discussed above, there are other limitations that may be classified as biological and experimental. Normal function gathered from *in vitro* assays does not guarantee there will be no functional defects *in vivo* (Watanabe et al., 2011). For example, functional results can be influenced by the isoform used (Tan et al., 2005; Wang et al., 2007), mRNA splicing, protein interactions that may not be present in heterologous expression systems (Watanabe et al., 2009), transcriptional factors that may impact gene and variant expression (Barc et al., 2022), and non-coding variants that may impact expression in native cells but not in the heterologous expression system. This can be addressed by studying variants in iPSC-derived cardiac myocytes, but at the expense of much lower throughput, (Sendfeld et al., 2019). Furthermore, though genotype-phenotype studies can identify LOF and GOF variants associated with specific diseases, there are variants that may overlap several diseases, such as in the case for *SCN5A*-E1784K and *CACNA1C*-E1115K (Makita et al., 2008; Kashiwa et al., 2023). Many of the assays developed to date have also not studied enough benign variant controls to be able to formally fulfil the ClinGen SVI working group criteria for validation of functional assays (Brnich et al., 2020). The strength of functional evidence for any given assay is dependent on i) the assay’s ability to accurately distinguish normal from abnormal function (Jiang et al., 2022) and ii) the number of control variants available to test. So, if there are very few benign missense variants (e.g., because the gene is very small) then it may not be possible to achieve more than moderate evidence strength (Jiang et al., 2022). Similarly, if a disease is very rare, then there may be an insufficient number of definitely (likely) pathogenic variants available to calibrate the assay to achieve more than moderate evidence strength. Furthermore, not all assays have incorporated the potential for dominant-negative effects, and difficulties in co-expression of multiple sub-units in the correct stoichiometry (Zou et al., 2022) need to be considered. There are also no assays yet developed for channels with more complex stoichiometries, e.g., L-type calcium channels which require co-expression of multiple subunits (Dolphin, 2016).

At an experimental level, temperature control can also be an issue, which is important to study ion channel kinetics of temperature-sensitive variants (Vandenberg et al., 2006; Abdelsayed et al., 2015; Lei et al., 2019; Jones, 2022; Ren et al., 2022; Kriegeskorte et al., 2023). Whilst temperature can be preset on most APC systems (e.g., Biolin Scientific, Fluxion and Nanion Technologies, Table 1), most APC assays for cardiac ion channels have been conducted at room temperature (Vanoye et al., 2018; Glazer et al., 2020b; Ng et al., 2021) due to an inherent instability of cell membranes at physiological temperatures leading to lower success rates. Temperature can have varying impacts on different properties. For example, increased temperature can exacerbate trafficking defects but also reduces inactivation in hERG channels but this may vary between variants. Analyzing the effect of temperature would also be especially valuable for fever-inducible disorders such as BrS (Dumaine et al., 1999; Keller et al., 2005; Peters et al., 2016) but at the moment this remains challenging for APC systems.

The widespread use of fluoride as the primary anion in internal solutions have benefited patch clamp studies through the formation of CaF_2_ crystals at the site of membrane rupture, improving the seal resistance and cell stability. In cardiac potassium channels, the use of fluoride did not alter biophysical properties and was shown to be significantly superior to alternatives, K-gluconate and KCl, for patch success rates (Zeng et al., 2008; Rapedius et al., 2022). Whilst in some neuronal sodium channels (Na_V_1.9, Na_V_1.7), fluoride has been reported to impact gating (Meadows et al., 2002; Rugiero et al., 2003; Coste et al., 2004; Jarecki et al., 2008) but not in others (Na_V_1.8) (Coste et al., 2004). The recent release of Fluoride-free APC chips could overcome these issues, however they are associated with a reduction of seal quality, e.g., from 77.9% with seal >1 GΩ to 35.9% > 1 GΩ in the fluoride free solutions (Rapedius et al., 2022).

## Clinical application of APC assays for personalized genomic medicine

### The genetic basis of cardiac channelopathies

Sudden cardiac death accounts for ∼80% of cardiac deaths in young, healthy individuals (Doolan et al., 2004; Bagnall et al., 2016). Approximately a third are from primary arrhythmogenic disorders (Doolan et al., 2004; Bagnall et al., 2016). Therefore, genetic tests can be incredibly valuable for variant discovery in arrhythmogenic diseases and for familial screening and therapy (Tester and Ackerman, 2007; Gladding et al., 2010). Identifying a variant, however, does not necessarily equate to finding the cause of the disease. An additional complication is that some diseases can be caused by variants in different genes (e.g., LQTS in *KCNQ1*, *KCNH2*, and *SCN5A*; Table 2) and management will vary depending on which gene is implicated (Wilde et al., 2022a). Robust APC assays can be useful to identify which variants alter function and are therefore most likely to be the true cause of disease.

**TABLE 2:** Summary of common cardiac channelopathy-associated genes. LQTS, Long QT Syndrome; CPVT, Catecholaminergic Polymorphic Ventricular Tachycardia. BrS: Brugada Syndrome; CCD, Cardiac Conduction Disease; SUDS, Sudden Unexplained Death Syndrome; SIDS, Sudden Infant Death Syndrome; ATS, Andersen-Tawil Syndrome; Dx, diagnostic; Px, prognostic; Tx, therapeutic/treatment; Recommended, indicated or useful (+++). Can be recommended or useful (++). May be considered or useful (+).

Phenotype			Impact of genetic testing^18^				Genotype			Functional evidence		
Disease	Prevalence	Dx yield	Dx	Px	Tx	Sub-type	Gene	Protein	Frequency	Mechanism	Assay available	Strength^29^
**LQTS**	1:2500^1^	30–80 (%) ^2,3,4,5,28^	+++	+++	+++	LQT1	*KCNQ1*	K_V_7.1	40–55 (%)	LOF *I*_Ks_	SyncroPatch^6,7,8^	N/A
											PatchXpress^9^	N/A
						LQT2	*KCNH2*	K_V_11.1	30–45 (%)	LOF *I*_Kr_	SyncroPatch^10,11,12,30^	(BS3_mod, PS3_mod)^12^ and (BS3, PS3)^30^
											DMS^13^	N/A
											MPRA^14^	N/A
						LQT3	*SCN5A*	Na_V_1.5	5–10 (%)	GOF *I*_Na_	SyncroPatch^15^	N/A
						LQT7/ATS	*KCNJ2*	K_ir_2.1	<1 (%)	LOF *I*_K1_		
						LQT8/TS	*CACNA1C*	Ca_V_1.2	<1 (%)	GOF *I*_Ca-L_	PatchXpress^16,^ ^a^	N/A
**BrS**	1:2000^19,20^	13–30 (%) ^3,4,22,27^	+	+	+	BrS1	*SCN5A*	Na_V_1.5	20–30 (%)	LOF *I*_Na_	SyncroPatch^15,17^	(BS3, PS3)^17^
											DMS^21^	N/A
**CPVT**	1:20,000^18^	35–47 (%) ^3,4,23^	+++	+	+	CPVT1	*RYR2*	RYR2	60–70 (%)	GOF	HEK293 Calcium imaging assays	N/A
										Ca^2+^ leak		
**PCCD**		>37 (%)^4,18^	+	+	+		*SCN5A*	Na_V_1.5	20 (%)	LOF *I*_Na_		
SQTS		14 (%) ^25^	+	+	+		*KCNH2*	K_V_11.1/hERG	<14 ^25^ (%)	GOF *I*_Kr_		

1 (Schwartz et al., 2009).

2 (Tester et al., 2006).

3 (Bai et al., 2009).

4 (Hofman et al., 2013).

5 (Lieve et al., 2013).

6 (Vanoye et al., 2018).

7 (Kuenze et al., 2020).

8 (Glazer et al., 2022).

9 (Trepakova et al., 2007).

10 (Ng et al., 2020).

11 (Ng et al., 2021).

12 (Jiang et al., 2022).

13 (Kozek et al., 2020).

14 (Ng et al., 2022).

15 (Glazer et al., 2020b).

16 (Balasubramanian et al., 2009).

17 (Ma et al., 2023).

18 (Wilde et al., 2022b).

19 (Barros et al., 2013).

20 (Vutthikraivit et al., 2018).

21 (Glazer et al., 2020a).

22 (Crotti et al., 2012).

23 (Medeiros-Domingo et al., 2009).

24 (Priori et al., 2002).

25 (Mazzanti et al., 2014).

26 (Gray and Behr, 2016).

27 (Kapplinger et al., 2010).

28 (Kapplinger et al., 2009).

29 (Brnich et al., 2020).

30 (Thomson et al., 2023).

^a^ APC, assay available but not optimized for disease.

In addition to finding variants in patients with clinical evidence for a genetic condition, there are an increasing number of patients who are having genome sequencing for other conditions and a rare, variant is identified in a cardiac ion channel gene. In theory, such variants could have the potential to significantly increase the risk of cardiac arrhythmias. In 2013, the ACMG devised a list of genes identifying highly penetrant genetic disorders amenable to medical intervention (Green et al., 2013). Secondary findings, defined as variants in the list of medically actionable genes that are determined to be disease-causing, are required to be reported irrespective of whether it was the original reason for seeking genetic testing (Kalia et al., 2017; Miller et al., 2021b). Regulated by the Secondary Findings Maintenance Working Group, the list currently contains 73 genes, of which 34 are associated with cardiovascular phenotypes including the aforementioned genes *KCNQ1*, *KCNH2* and *SCN5A* (Miller et al., 2021a).

### Bench to bedside: variant of uncertain significance

Variant classifications are guided by ACMG/AMP’s evidence strength-dependent, multi-tiered criterion (Richards et al., 2015) and have been adopted by 95% of laboratories (Niehaus et al., 2019). Classifications are used to determine clinical risks however, a limitation of this system is when insufficient clinical phenotype data are available to determine pathogenicity (Kroncke et al., 2020). ClinVar contains interpretations for more than half a million variants (Landrum et al., 2018; Landrum et al., 2019). However, concordance rates for cardiac genes was only 62% (Amendola et al., 2020) and approximately half of all variants uncovered in genes implicated in inheritable cardiac disease are classified as VUS (Anderson et al., 2022). Unfortunately, the classification where there is most concordance is VUS and, alarmingly, discordances that would influence clinical recommendations were found in 11% of variants when the Sequence and Diagnostic Yield working group evaluated variant classifications in medically actionable genes across 8 laboratories (Amendola et al., 2020). Functional assays have the potential to help resolve much of this discordance. However, as noted by Harrison and colleagues (Harrison et al., 2017), there has been very inconsistent application of evidence from functional assays between genetic testing laboratories. Consequently, a detailed ClinGen framework for the application of functional evidence was released (Table 3) (Brnich et al., 2020) and it has been shown that high throughput APC functional assays for cardiac ion channels can be designed to meet a strong level of evidence for assessment of variants (Jiang et al., 2022; Ma et al., 2023; Thomson et al., 2023).

**TABLE 3:** The level of evidence applicable for each functional assay is determined by an ‘Odds of Pathogenicity’ score (Reproduced from Brnich et al. (2020), licensed under CC-BY 4.0). Functional evidence BS3: well established functional evidence shows no deleterious effect. PS3: Well-established functional studies show a deleterious effect (Richards et al., 2015).

$OddsPath=\frac{\left[P_{2}*\left(1-P_{1}\right)\right]}{\left[\left(1-P_{2}\right)*P_{1}\right]}$	
Odds of Pathogenicity (OddsPath)	Evidence strength equivalent
<0.053	BS3
<0.23	BS3_moderate
<0.48	BS3_supporting
0.48–2.1	Intermediate
>2.1	PS3_supporting
>4.3	PS3_moderate
>18.7	PS3
>350	PS3_very_strong

## Future of functional assays for cardiac ion channel genes

### APC assays for multi-subunit complexes

For many ion channels, functional activity requires co-expression of multiple subunits. It is possible to co-express two subunits from a single plasmid by including an Internal Ribosome Entry Site (IRES) between the two cDNA sequences. This approach has been used to co-express beta β-subunits with α- or other β-subunits for sodium (Fletcher et al., 2011; Nakajima et al., 2015) and potassium channels (Ng et al., 2020; Ng et al., 2021). However, the expression between the genes using IRES may not be optimal as the gene located after the IRES is typically expressed at lower levels (Mizuguchi et al., 2000; Bochkov and Palmenberg, 2006). Recently, a modified Flp-In HEK293 was developed to enable the expression of 2 genes at equal expression by introducing a second plasmid that has puromycin antibiotic resistance (in addition to hygromycin on the first plasmid) (Ward et al., 2011; Jensen et al., 2020). By incorporating IRES into this modified Flp-In HEK293, up to 4 genes can be expressed in a single Flp-In HEK293 cell line. This will facilitate the efficient generation of multi-subunit channel complexes, such as the L-type calcium channel (Ca_V_1.2, *I*_Ca,L_) which is crucial for the plateau phase of the cardiac action potential and requires co-expression of *CACNA1C*, *CACNB2B*, and *CACNA2D1*. Ca_V_1.2 GOF variants can cause Timothy Syndrome, a multi-organ dysfunction associated with LQTS (LQTS8) and sudden cardiac death (Splawski et al., 2004). While Ca_V_1.2 LOF variants have been associated with BrS (1%–2%) (Gray and Behr, 2016; Cerrone et al., 2022; Nakano and Shimizu, 2022) and SQTS (Templin et al., 2011), although this association is not yet classified as definitive (Wilde et al., 2022b). This strategy can also be employed to generate stable cell lines that co-expressed both *KCNQ1/KCNE1* and the A-kinase adaptor protein, *AKAP9,* which is necessary to enable modulation by adrenergic signaling (Dvir et al., 2014).

### Companion diagnostics

In addition to using APC to determine the effect of variants on channel function, it is possible to simultaneously test the effect of drugs on each variant. This approach is likely to be particularly important for GOF mutations. For example, a recent *in vitro* study comparing the efficacy of sodium channel blockers found phentytoin, an anti-seizure medication, to be more effective at rescuing the LQT3 phenotype than the commonly recommended medication, mexiletine, for *SCN5A-*F1760C (Stutzman et al., 2023). Similarly, one can also envisage screening GOF *CACNA1C* variants in Timothy syndrome patients to identify the optimal drug for suppressing the late *I*_Ca,L_ without reducing peak *I*_Ca,L_. This approach, however, need not be limited to GOF variants. For example, APC methods have provided important preliminary screening data to identify LOF variants in *CFTR* that are amenable to rescue with Trikaftor (Brüggemann et al., 2017).

## Conclusion

In recent years there has been tremendous progress in developing high throughput APC assays to assess the functional effects of cardiac ion channel variants. One can envisage that within a few years all presently known clinically occurring variants in these genes will have been characterized. Whilst it is unlikely that APC assays will enable prospective characterization of all potential missense variants in an ion channel, this could be achieved with higher throughput multiplexed assays of variant effect (MAVE) assays (Findlay et al., 2018), including, e.g., fluorescence based trafficking assays and cell survival assays combined with deep mutational scanning (Glazer et al., 2020a; Kozek et al., 2020; Fayer et al., 2021; Coyote-Maestas et al., 2022; Ng et al., 2022). In this context, APC assays have been very useful for validating the results from these techniques (Glazer et al., 2020a; Kozek et al., 2020; Ng et al., 2022). Ultimately, this could facilitate the development of databases containing functional data for all potential variants that clinicians could access immediately upon finding any new variants in an ion channel gene. One could also envisage APC functional genomics assays being combined with drug screening to enable identification of optimal drug therapy for each specific variant.
